# ROS and Brain Diseases: The Good, the Bad, and the Ugly

**DOI:** 10.1155/2013/963520

**Published:** 2013-12-05

**Authors:** Aurel Popa-Wagner, Smaranda Mitran, Senthilkumar Sivanesan, Edwin Chang, Ana-Maria Buga

**Affiliations:** ^1^Department of Psychiatry, Rostock University Medical School, Gehlsheimer Straße 20, 18147 Rostock, Germany; ^2^Center of Clinical and Experimental Medicine, University of Medicine and Pharmacy, 200349 Craiova, Romania; ^3^Department of Biomedical Sciences, College of Veterinary Medicine, Ames, IA 50011, USA; ^4^Stanford University School of Medicine, MIPS, Canary Center at Stanford for Cancer Early Detection, CA 94304, USA

## Abstract

The brain is a major metabolizer of oxygen and yet has relatively feeble protective antioxidant mechanisms. This paper reviews the Janus-faced properties of reactive oxygen species. It will describe the positive aspects of moderately induced ROS but it will also outline recent research findings concerning the impact of oxidative and nitrooxidative stress on neuronal structure and function in neuropsychiatric diseases, including major depression. A common denominator of all neuropsychiatric diseases including schizophrenia and ADHD is an increased inflammatory response of the brain caused either by an exposure to proinflammatory agents during development or an accumulation of degenerated neurons, oxidized proteins, glycated products, or lipid peroxidation in the adult brain. Therefore, modulation of the prooxidant-antioxidant balance provides a therapeutic option which can be used to improve neuroprotection in response to oxidative stress. We also discuss the neuroprotective role of the nuclear factor erythroid 2-related factor (Nrf2) in the aged brain in response to oxidative stressors and nanoparticle-mediated delivery of ROS-scavenging drugs. The antioxidant therapy is a novel therapeutic strategy. However, the available drugs have pleiotropic actions and are not fully characterized in the clinic. Additional clinical trials are needed to assess the risks and benefits of antioxidant therapies for neuropsychiatric disorders.

## 1. Introduction

The earth began its life without free oxygen in its atmosphere [1]. Oxygen accumulation is a consequence of the establishment and propagation of photosynthesizing archea and bacteria on this planet [2]. With the arrival of the world's first de facto pollutant (i.e., oxygen), approximately 3 billion years ago there evolved organisms that reductively metabolized oxygen to produce ATP in mitochondria [3] (i.e., aerobic respiration). Mitochondrial energy metabolism yields several reactive oxygen species (ROS) including oxygen ions (O_2_^−^, the primary ROS), free radicals, and peroxides (inorganic and organic). The presence of ROS produced profound consequences for life on earth, both beneficial and deleterious. For example, a wealth of evidence suggests that high levels of ROS are intimately linked to the appearance of neuronal death in various neurological disorders. These include chronic diseases (Parkinson's disease or Alzheimer's disease) [4], acute injury of the brain (brain trauma and cerebral ischemia) [5, 6], or psychiatric disorders (autism, attention deficit hyperactivity disorder, depression, and schizophrenia) [7].

**Figure 1:**
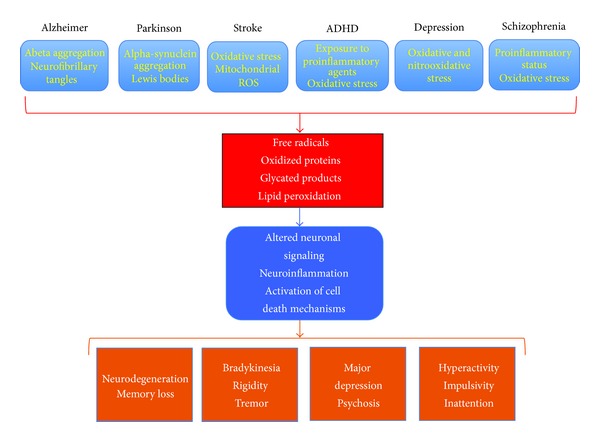
Schematic representation of oxidative stress-related mechanisms underlying disease development in Alzheimer's disease (AD), Parkinson disease (PD), stroke, attention deficit and hyperactivity disorders (ADHD), schizophrenia, and depression.

An increase in oxidative and nitrooxidative stress and a decrease in the antioxidant capacity of the brain are key factors involved in the etiology of neuropsychiatric diseases (Figure 1). In the following we will detail both the beneficial and deleterious impacts of these Janus-faced processes.

## 2. ROS Are Required for Physiological Processes

Even though ROS are involved in a number of diseases, they are also very pertinent mediators of several normal physiological processes. All of the good ROS are products of turnover in the mitochondrial respiratory chain. The highly reactive nature of singlet oxygen can even be exploited to make reactive peroxides that can serve as antimicrobial agents [8]. Most of the physiological effects are in fact mediated by reactive oxygen species (ROS) derivatives of superoxide. Similarly, the superoxide anion (O_2_^−^^∙^), through its derivative, the hydroxyl radical (∙OH), plays an essential role in cell physiology by stimulating the activation of guanylate cyclase and formation of the “second messenger” cGMP in cells and activation of the transcription factor nuclear factor kB (NF-kB) by hydrogen peroxide in mammalian cells [9]. Under normal physiological conditions, the NO radical (NO∙) regulates the vascular tone by smooth muscle relaxation.

In the inflammatory response, macrophages and neutrophils are attracted by activated T lymphocytes and IL-2 and produce high levels of O_2_^−^, which along with other ROS destroy the engulfed bacteria *(oxidative burst) *[10]. In brain tissue, ROS are generated by microglia and astrocytes and modulate synaptic and nonsynaptic communication between neurons and glia. ROS also interfere with increased neuronal activity [11] by modifying the myelin basic protein and can induce synaptic long-term potentiation, a form of activity-dependent synaptic plasticity and memory consolidation. Furthermore, results from animal models suggest that the role of O_2_^−^ in modulating synaptic plasticity, memory formation, and learning is altered by age [12].

## 3. Deleterious Effects Associated with ROS

Activated oxygen species, especially those that share electronic orbital features with singlet oxygen, are highly reactive, and therefore the evolutionary adaptation by organisms to make oxygen wholly beneficial has never been complete. Consequently, there are many deleterious effects associated with excessive, activated ROS. ROS are thereby capable of producing membrane damage, changes in the inner proteins affecting their structure and function, lipids denaturation, and structural damage to DNA. In the brain, those effects appear when ROS are in excessive concentrations and thus the ability of antioxidant mechanisms of neurons to counterbalance the damaging reactions is diminished [13].

The brain is especially susceptible to the assaults perpetrated by ROS. This is because the brain as an organ is a major metabolizer of oxygen (20% of the body consumption) and yet has relatively feeble protective antioxidant mechanisms. Thus it is especially vulnerable to oxidative stress. The brain contains a large amount of polyunsaturated peroxidizable fatty acids along with high levels of iron that act as a prooxidant and sometimes induce autophagic cell death in hemorrhagic stroke. Synaptic transmission involving dopamine and glutamate oxidation may also occur in the brain [13–15]. In addition, lipid peroxidation leads to the production of toxic compounds such aldehydes or dienals (e.g., 4-hydroxynonenal) which may cause in turn neuronal apoptosis [16].

At the DNA level, oxidative modifications may cause rapid depletion of intracellular energy by activating DNA repair enzymes. Energy scarcity after stroke will cause in turn endonuclease-mediated DNA fragmentation, the key mechanism that leads to DNA-damage [17].

## 4. Brain Mitochondrion and Oxidative Stress

The mitochondria is the cellular powerhouse that generates ATP and therefore is a key participant in all physiological and pathological events. Following ischemia-associated excitotoxicity and other neurodegenerative insults, mitochondria will take up calcium which leads to an increased production of reactive oxygen species (ROS). As a result, neuronal cells face excessive amounts of ROS [18]. The mechanisms linking mitochondrial calcium uptake and ROS production are incompletely understood but may involve an increase in the permeability of the mitochondrial membranes to small molecules and thereby initiate cell death through release of apoptosis-inducing factors via the opening of an as yet unidentified megapore protein [18–21].

Once opened, the pore can cause the collapse of the proton gradient. This promotes collapse of respiratory control as well as release of Ca^2+^ stored in the mitochondrial matrix. Furthermore, since the mitochondrial matrix is negatively charged, K^+^ can enter through the pore and cause osmotic swelling [22]. A bioenergetics failure ensues due to the inhibition of oxidative phosphorylation and ATP synthesis. The few ATP molecules generated by glycolysis or by residual functional mitochondria are hydrolyzed by ATP synthase that work in reverse under such extreme conditions [22]. If the pore remains open for a long period major consequences for energy metabolism and even cellular death may occur.

An increasing number of studies report the prevalence of oxidative stress and mitochondrial abnormalities in numerous neuropsychiatric disorders [23–30]. A spectrum of oxidative stress markers involved in schizophrenia and anxiety related disorders were thoroughly categorized based on several biochemical assays [31, 32] as well as genetic assessment studies [32]. Recent genetic studies pinpoint at mutations in several genes encoding proteins required for synaptic plasticity suggesting a plausible link between neuropsychiatric diseases and neurodevelopmental abnormalities [33, 34]. Notably, mutations in PTEN-induced kinase 1 (PINK1) gene function have been attributed to oxidative stress and mitochondrial fragmentation [35]. However, alterations in GMP-PKG signaling leading to increased brain phosphodiesterase-2 (PDE2) levels [26, 36, 37] and NOX2-dependent mechanisms [38] are believed to drive oxidative stress-related events in the pathogenesis of psychiatric disorders.

Defects in the electron transport chain (ETC) system and especially the mitochondrial complex dysfunctions have been reported to be involved in autism [39, 40] and schizophrenia [40]. Additionally, defects in pyruvate dehydrogenase activity and copy number variations in mtDNA were also seen in autistic subjects [40]. In a more recent work, involving a transgenic mouse model that expresses the gene, “putative dominant-negative disrupted in schizophrenia 1” (DN-DISC1), a plausible mechanistic link between prefrontal oxidative stress and neuropsychiatric problems was explored [41]. Likewise, more recent genetic studies point to alterations in a set of genes associated with epigenetic mechanisms and oxidative stress pathology thereby suggesting histone modifications and DNA methylation in schizophrenia related psychopathology [42].

## 5. Oxidative Stress Mediated Inflammation and Neuropsychiatric Diseases

Recent findings suggest that neuroinflammation is an important player in the pathophysiology of neuropsychiatric diseases such as stroke, depression, Alzheimer's diseases, or schizophrenia [43, 44].

### 5.1. Cerebral Ischemia (CI)

After the onset of cerebral ischemia, oxidative stress plays a major role in neuroinflammatory reactions [45, 46]. In postischemic brains, oxidative stress triggers activation of microglia and astrocytes [47] leading to striking elevations in the levels of inflammatory mediators such as cytokines, chemokines, and matrix metalloproteases [48] and causing the loss of endothelial cell integrity in the brain as manifested by upregulation of cell adhesion molecules and neutrophil infiltration [49]. This probably provokes subsequently secondary inflammation and glial immune response resulting in permanent brain cell damage [48].

Redox-mediated increase in free radicals in the postischemic brains notably leads to augmented expression of several proinflammatory genes whose expression is mediated through the transcription factor nuclear factor-kappa-B or NF-*κ*B [48]. Importantly, NF-*κ*B mediated proinflammatory reactions and innate immune responses are prominent features in cerebral ischemic conditions [50–52]. Although activation of innate immunity by Toll-like receptors seems to promote regenerative mechanisms [53], neuronal loss critically involves ROS induced TLR-mediated inflammatory responses during cerebral ischemia [54–57].

### 5.2. Parkinson's Disease

In Parkinson's disease (PD), a considerable body of evidence suggests that both dopaminergic and nondopaminergic cells undergo degeneration [58–60]. The innate immune response seen in the CNS of PD subjects involves a spectrum of oxidative stress and inflammatory responses that cause neurodegeneration. However, the key loss of dopaminergic neurons involves oxidative stress and neuroinflammatory mechanisms through increased levels of inducible nitric oxide synthase (iNOS) followed by activated microglia, T-cell infiltration, and astrogliosis leading to accumulation of O_2_^−^ and NO free radicals [59]. Dopaminergic neuronal loss via oxidative stress-mediated inflammation also involves cyclooxygenase-2 (COX2) overexpression [59]. Additionally, higher levels of myeloperoxidase, generated by reactive astrocytes, could even elevate the levels of reactive (∙OH) and NO_2_^−^ radicals that could in turn lead to neuronal loss in Parkinson's disease [59]. Similarly, the upregulation of NADPH-oxidase, which is known to be associated with inflammation and ROS generation, in PD has been tied to microglial activation, local ROS elevation, and subsequent dopaminergic neuronal loss [61].

The involvement of microglial activation most likely attracts lymphocytes to the site of injury. Subsequent pathophysiological sequelae involve damage to the integrity of the blood brain barrier (BBB), and then increased shedding of several inflammatory cytokines into the cerebral spinal fluid (CSF) of PD patients [62, 63]. As reactive microgliosis seems to be a major event in PD pathogenesis, altered cytokines and ROS levels in microglia [64] as well as augmented microglial NADPH-derived ROS accumulation [65] all contribute to the neurotoxicity seen in PD.

The research findings from Reynolds and colleagues implicate the role of adaptive immunity functions during microglial inflammation in Parkinson's disease [66]. On the other hand, the effector T-cell (E) responses are believed to be the mediators of microglial activation and subsequent neurodegeneration. Taken together, both innate and adaptive immunity play consistent, prominent roles in causing neuronal death in PD.

### 5.3. Alzheimer's Disease (AD)

Major events associated with oxidative stress could exacerbate inflammation-related pathologies in Alzheimer's disease (AD) [67–69]. Recent reports suggest that oxidative stress-mediated binding of advanced glycation endproduct (AGE) to its receptor (RAGE) can adversely increase the microglial inflammation and cytokine release. Such events contribute to neuronal loss in AD [70]. In AD patients, both reactive astrocytes and microglial activation are involved in amyloid beta protein-mediated oxidative stress and inflammation [71–73] as well as compromised immune responses [71] within the cerebral parenchyma. Recent genomic studies [74] in AD patients confirmed previous observations that showed upregulation of several pathways associated with the innate immune system following microglia activation and inflammation in aging. The researchers concluded that such findings would ultimately account for neurodegeneration and memory loss in AD.

The important role conferred to Toll-like receptors (TLR2 and TLR4) in AD as peripheral biomarkers [75] of AD pathophysiology [75–77] is well studied. Notably, the part played by TLR receptors in AD progression addresses the importance of innate immune responses [78]. However, the striking elevation of TLR4 expression in neurons by the toxic action of Abeta 1–42 or by increased elevation of the lipid peroxidation product, 4-hydroxynonenal (HNE), obviously suggests that oxidative stress does drive neuronal loss in AD [77].

### 5.4. Multiple Sclerosis

The demyelination and axonal damage triggered by oxidative stress and inflammatory reactions are widely reported in multiple sclerosis (MS) [79]. The subsequent appearance of peroxynitrate molecules is believed to inflict severe damage to brain cells and especially neurons [80]. Recently, both ROS and RNS (reactive nitrogen species) compounds are considered to be the early causative factors for the inflammatory reactions associated with MS. This subsequently leads to considerable loss of oligodendrocytes and axons [81]. Moreover, the early mitochondrial damage caused by oxidative stress seems to be critical for subsequent inflammatory processes and neuronal loss in multiple sclerosis [82].

### 5.5. Neurodevelopmental Disorders

Epidemiological studies on prenatal exposure to infection during the developmental period suggests that the immune system plays an important role in a number of disorders such as schizophrenia, depression, or cognitive impairment [83]. The innate immune system triggers a cascade of events that results in an increased synthesis of cytokines, interferons, and other immunological mediators thereby altering the balance of pro- and anti-inflammatory cytokines. Since cytokine receptors are expressed as early as the fetal period, it is thought that inflammatory cytokines may represent a key mechanism involved in neurodevelopmental disorders [84, 85]. Indeed, recent studies have shown that prenatal exposure to proinflammatory agents like Il–6, Il–8, and TNF alpha can increase the risk for diseases like autism or schizophrenia by inducing both neuromorphological and neurochemical changes in the immature brain [86–88].

The vulnerability to develop psychiatric diseases later in life (fetal programming) involves not only the immune response of the brain, but also the changes in glial cell function [89, 90]. Thus, assessment of microglia in postmortem autism cases revealed a strong microglial cortical activation suggesting that activated microglia may play a central role in the pathogenesis of autism [91].

### 5.6. Depressive Disorders

Major depressive disorders are often associated with an increased inflammatory response of the brain. A study employing the serotonin transporter (SERT) mutant rats has shown that deletion of the SERT gene is associated with an increased level of proinflammatory cytokines and an increased number of activated microglia suggesting that the serotonin transporter dysfunction plays an important role in the pathogenesis of depressive disorders via an increased glial response to inflammatory agents [92, 93]. More specific, Macchi and colleagues [93] showed that the SERT mutant rats displayed an activated microglial phenotype under basal conditions. The mechanisms for microglial activation and its consequences are still not completely understood. However, several studies investigating the role of proinflammatory cytokines like tumor necrosis factor (TNF) alpha or interleukins (IL1 beta, IL6, and IL8) have involved microglial activation in schizophrenia pathogenesis [94, 95].

The progression of bipolar disorders is multifactorial and include the interaction between the neurotransmitters, neuropeptides, oxidative and nitrosative stress and cytokines, and neurotrophins including BDNF [96, 97]. Most importantly, emerging evidence in animal models and also in humans suggests that anti-inflammatory therapy may delay the progression of the diseases [98].

## 6. Biomarkers of Oxidative Stress in Neuropsychiatric Diseases

The levels of several growth factors and metabolites are altered in cerebral tissue, cerebrospinal fluid (CSF), and serum of patients exposed to oxidative stress and can be used to assess risk factors or as potential biomarkers. Similarly, longitudinal *in vivo* ^1^H-MRS that was used to evaluate the postinjury evolution of 20 individual neurochemicals after traumatic brain injury (TBI) revealed a dramatic depletion of glutathione (GSH) and ascorbate (Asc), two endogenous antioxidants with the highest concentration in the central nervous system [99]. The following sections give an account of current biomarkers of oxidative stress in neuropsychiatric diseases.

### 6.1. Developmental Disorders

Although many of the cognitive and behavioral features of autism spectrum disorders (ASD) are associated with dysfunctions of the central nervous system, ASD also appears to be associated with systemic metabolic abnormalities such as oxidative stress and mitochondrial dysfunction [100–102]. Glutathione deficiency also contributes to abnormalities seen in autistic behavior [40, 103] as well as schizophrenia and bipolar disorders [104]. Likewise, examination of urinary samples from schizophrenia subjects revealed considerable alterations in 8-oxo-7,8-dihydro-20-deoxyguanosine and 8-oxo-7,8-dihydroguanosine levels indicating oxidative stress induced nucleic acids damage [105]. Similarly, in plasma F2-dihomo-isoprostanes, a specific component of myelin in the brain of primates, plasma represents an early marker of Rett syndrome, a developmental disorder that almost exclusively affects females but has been found in male patients, too [106].

A recent meta-analysis of oxidative stress in schizophrenia suggests that red blood cells (RBC) SOD levels and to a lesser extent RBC catalase and plasma nitrites, were significantly decreased in acutely relapsed inpatients, first-episode psychosis, and stable medicated outpatients [107].

Several markers of lipid peroxidation including the ubiquitous lipid peroxidation product, malondialdehyde (MDA), have been found to be significantly increased in ADHD patients. The study comprised 20 adult-ADHD patients and 21 healthy volunteers, to whom malondialdehyde levels were measured in plasma. The study evaluated indirectly the level of ROS and found that the mean plasma MDA levels of patients having adult-ADHD were significantly higher than those of the control group. The results suggest a relation between oxidative metabolism and adult ADHD [108].

### 6.2. Depressive Disorders

Studies conducted on postmortem tissue from depressed patients have shown reduced levels of hippocampal, an anatomical structure involved in anxiety and depressive behaviors, as well as cortical BDNF [109]. In addition, a polymorphism in the BDNF gene has been associated with depression [110–113].

In the periphery, a meta-analysis provided strong evidence that BDNF levels in serum of depressed subjects are lower as compared to those of the healthy controls. Further, the antidepressant treatment restored BDNF to normal levels [114].

After adipose tissue, the organ with the highest lipid content is the brain and elevated serum levels of lipid peroxidation products have been often reported in bipolar disorders. Because the axonal membranes and myelin sheaths of the brain are rich in lipids, it has been proposed that lipid peroxidation products may be promising peripheral biomarkers of underlying white matter abnormalities in bipolar disorders (BP). A recent study combined diffusion tensor imaging and biochemical analysis of serum to examine relationships between measures of white matter integrity as measured by fractional anisotropy, radial diffusivity, and peripheral measures of lipid peroxidation, the lipid hydroperoxides, LPH and 4-hydroxy-2-nonenal, 4-HNE. The study suggests that serum LPH may be useful in the development of a clinically relevant assay for peripheral biomarkers of BD [115].

Similarly, lipofuscin granule accumulation has been described in biopsies from patients suffering from major depressive disorder (MDD) indicating the impact of oxidative stress on neurovascular abnormalities in these patients [116].

### 6.3. Alzheimer Disease

Mitochondrial dysfunction can also be detected in peripheral tissues. Although erythrocytes are considered as passive “reporter cells” for the oxidative status of the whole organism, decreased glutathione peroxidase activity (GPx) in RBC may be considered as a new peripheral marker for AD [117]. Several other studies showed a decreased cytochrome c activity and increased levels of ROS in human platelets from AD patients [118–120]. Similarly, lymphocytes of AD patients are characterised by increased basal ROS levels [121].

Other classical markers of oxidative stress in plasma of early stage AD patients include thiobarbituric acid reactive substances (TBARS) as an index of lipid peroxidation, protein carbonyl content as an index of protein oxidation, the enzymatic activities of GPx, catalase (CAT), and superoxide dismutase (SOD) as well as the plasma levels of total glutathione (reduced GSH plus oxidized glutathione (GSSG) [122].

### 6.4. Parkinson's Disease

Similarly, 4-hydroxynonenal (4-HNE), an alpha, beta-unsaturated hydroxyalkenal produced by peroxidation of polyunsaturated fatty acids, is widely recognized as a specific marker of oxidative stress both centrally and in the CSF and serum of PD patients [123–127].

Likewise, several markers of lipid peroxidation including the ubiquitous lipid peroxidation product, malondialdehyde (MDA), have been found to be significantly increased in PD brains [128–131]. There is also strong evidence for lipid peroxidation products like hydroxyeicosatetraenoic acid in plasma, leukocytes, and erythrocytes from PD patients [132–134]. Finally, higher nitric oxide and peroxynitrite serum levels have been recently reported in Parkinson's disease versus controls [135].

## 7. Targets of Therapy in Neuropsychiatric Disorders

Oxidative stress and neuroinflammation are key factors contributing to aging of the brain. Many studies highlighted the importance of antioxidative defenses in the aging process [22, 136, 137], but it is still unclear whether these responses are beneficial or detrimental. A diminished endogenous antioxidative capacity of the brain can promote aged-related diseases such as cerebral ischemia or neurodegenerative disorders. In order to protect the brain from both aged-associated pathological diseases and the aging process itself, it becomes imperative to understand the molecular and cellular mechanisms of antioxidative and/or inflammatory response.

### 7.1. Activators of the Nrf2/ARE Pathway

The nuclear factor erythroid 2 related factor (Nrf2) is a dimer of the p45 protein and a member of the small Maf family proteins involved in the modulation of both antioxidant and anti-inflammatory signaling [138].

As cells are permanently exposed to a variety of oxidative stressors they must be able to trigger antioxidative signaling pathways in order to maintain redox homeostasis. To this end Nrf2 is activated, via phosphorylation, while under oxidative stress (such as in hypoxia/ischemia reperfusion or in neurodegenerative diseases) it is shuttled to the nucleus where it builds up a dimer with the small Maf. The activated complex in turn promotes the transcription of genes involved in neuroprotection. An *in vitro* study on primary cortical cultures has recently shown that prolonged expression of the transcription factor NF-E2-related factor 2 (Nrf2) induced by hypoxia and oxidative stress acts neuroprotectively against oxygen glucose deprivation. By inserting the Nrf2 gene in an inducible gene construct, a controlled, neuroprotective effect can be achieved by overexpressing Nrf2 not only during hypoxia but also after reperfusion [139].

The key trigger to this neuroprotective cascade is the binding of Nrf2 to the antioxidant response elements (AREs) [140–142]. Therefore, exogenous Nrf2/ARE activators may represent powerful drugs to activate the antioxidant and defensive acting genes. The Nrf2/ARE pathway can be pharmacologically activated both by natural products such as sulforaphane [143, 144], polyphenols, epigallocatechin 3-gallate (EGCG), and curcumin [145] and synthetic drugs including triterpenoids and N-(4-(2-pyridyl)(1,3-thiazol-2-yl))-2-(2,4,6-trimethylphenoxy) acetamide, known as CPN-9 [146].

Interestingly, recent studies have also shown that Nrf2/Hmox activation may enhance cell proliferation and survival in the subventricular zone (SVZ) of aged brains by reverting microglial phenotype into the proneurogenic phenotype [147, 148]. We also have observed that during conditions of cerebral ischemia, the aged brain upregulates the Hmox1 gene only partially and later (day 14 after-ischemia) as compared to young animals which activate this gene strongly and early (day 3 after-infarct) after stroke [149].

### 7.2. Biochemicals: Omega-3 Polyunsaturated Fatty Acids and Vitamins

Of these, omega-3 polyunsaturated fatty acid (PUFA) is one of the few compounds that are able to modulate the expression of genes involved in cell signaling, division, apoptosis, and oxidative stress [150]. Mounting evidence indicates that fatty acid deficiencies or imbalances may also contribute to a range of adult psychiatric and neurologic disorders including ADHD, DCD (developmental coordination disorder), and autism.

A recent study investigated the antioxidant effect of alpha linolenic acid in adults and children with ADHD, DCD, and autism. Omega-3 polyunsaturated fatty acids were given as supplements to patients diet, and the changes in hyperactivity, attention, and other disruptive behaviors were analyzed. The patients who received flax oil (an oil rich in 18 carbon omega-3 fatty alpha-linolenic acid) did show a significant improvement in the symptoms of ADHD reflected by a reduction in total hyperactivity scores [151]. However, the diet with mixed omega-3 and omega-6 supplementation had only a modest effect on attention and hyperactivity symptoms in 117 children with DCD. The treatment did not show a significant effect on motor skills but did show a significant improvement in reading, spelling, and behavior versus placebo during the 3 months of treatment [152].

Vitamins have also been used in clinical trials. A study was performed on patients over 70 years old, who were diagnosed with dementia and other cognitive dysfunctions [153]. An improvement in their cognitive performance was observed, after vitamin C and E were given as food supplements. Of note, a positive response was seen in vascular dementia but not in Alzheimer's disease [153]. In other studies, long-term high dosing of vitamin E supplementation increased the risk of hemorrhagic stroke and other causes of mortality, raising questions about the benefit or the harm of this treatment [154]. The presence of vitamin D seems to regulate autophagy that is used by the cell to degrade cytosolic macromolecules and organelles in the lysosome. Being an adaptive response, the autophagy can be useful or deleterious depending on the energetic status of the cell [155, 156].

## 8. Peroxisome Proliferator-Activated Receptor-Gamma Agonists

In human and animal models, the cognitive decline in vascular dementia is dependent on the hippocampal function. Recently, peroxisome proliferator-activated receptor gamma (PPAR-gamma) agonists were shown to diminish oxidative stress, inflammation, and apoptosis in the central nervous system [157, 158]. Peroxisome-proliferator activator receptor-gamma is a nuclear receptor with a key role in energy homeostasis and inflammation that has been implicated in the oxidative stress response.

Telmisartan is a special angiotensin II receptor blocker (ARB) and a partial agonist of the (PPAR-gamma). A recent study asked if telmisartan protects against cognitive decline in a rat model of vascular dementia. Indeed, it was found that telmisartan acts neuroprotectively against cognitive decline after cerebral ischemia by promoting anti-inflammatory and antioxidant effects [159, 160]. In particular, it has been hypothesized that ARB might act neuroprotectively by upregulating the levels of BDNF, a known antioxidant, in the hippocampus. Indeed, after 28 days of treatment with telmisartan, BDNF expression in the hippocampus was significantly higher as compared to controls and the animals showed an improved cognitive performance. It was inferred that telmisartan may protect the cells via upregulation of BDNF and its receptor TrkB, in the hippocampus possibly via angiotensin II-induced anti-oxidative stress [161]. Recently, another PPAR-gamma agonist, 15d-PGJ2, has been shown to exert neuroprotection by inhibiting neuronal autophagy in stroke model [162].

Thiazolidinediones (TZDs) are still another potent synthetic agonists of PPAR-gamma that have been successfully used to diminish inflammation after cerebral ischemia [163]. Similarly, pioglitazone and rosiglitazone are two TZDs with proven efficacy in reducing inflammation and upregulating antioxidant enzymes after spinal cord injury [164]. Recently, the neuroprotective efficacy of a TZD-unrelated PPARgamma agonist L-796,449 has been tested in an animal model of stroke. The study showed that L-796,449 decreased the infarct size and improved neurologic outcomes [165].

### 8.1. Gases: Hyperbaric Oxygen and H_2_S

The first study that used hyperbaric oxygen therapy to treat autistic children showed a significant level of improvement of autistic symptoms in 75% of patients [166]. The results have been confirmed in a second study that evaluated social development, fine motor and eye-hand coordination, language development, gross motor development, and self-help skills, before and after the treatment of children with autism. It should be noted that the beneficial effect of hyperbaric oxygen for brain diseases has been previously shown by D. A. Rossignol and L. W. Rossignol in 2006 who showed that hyperbaric oxygen therapy improved oxygen levels in ischemic area by increasing the oxygen concentration in plasma as a compensatory mechanism in hypoxia [167].

Hydrogen sulfide (H_2_S) is another gas recently used in several neuroprotective studies. H_2_S is a mild inhibitor of oxidative phosphorylation and can protect neurons after a stroke [168]. The study was performed on 17-month-old male Sprague-Dawley rats, and focal cerebral ischemia was induced by reversible occlusion of the right middle cerebral artery. Exposure of poststroke rats to a mixture of air and hydrogen sulfide for 2 days resulted in deep and sustained hypothermia (31.8 ± 0.7°C). An improvement in the post-stroke recovery of complex sensorimotor skills along with a 50% reduction in infarct size was noted. There were no obvious physiological side effects. Hypothermia resulted in a reduction in the number of phagocytic cells as well as decreased transcriptional activity of several genes related to inflammation and apoptosis including caspase 12, NF-kappa B, and grp78 in the peri-infarcted region [168].

Two further studies showed that indeed hydrogen-rich saline can benefit the brain in a *global cerebral* ischemia/reperfusion model (four-vessel occlusion model) and in a rat model with permanent *focal cerebral* ischemia (permanent middle cerebral artery occlusion), respectively [169, 170]. The results demonstrated that intraperitoneal injection of hydrogen-rich saline offers strong neuroprotective effects by reducing oxidative stress and inflammation. Thus, the level of endogenous antioxidant enzymes (superoxide dismutase-SOD and catalase-CAT) was increased, whereas the concentration of oxidative products (8-iso-PGF2*α* and malondialdehyde) and inflammatory cytokines (TNF-*α* and IL-6) was decreased [169, 170].

### 8.2. Metals: Lithium

Healthy volunteers treated with lithium for a period of 2–4 weeks showed decreased superoxide dismutase levels and superoxide dismutase/catalase ratio as well as diminished hydrogen peroxide concentrations. Therefore, lithium has a potential role for neuroprotection in bipolar disorders and even in neurodegenerative diseases. Lithium seems to be the gold standard due to its ability to prevent/or reverse DNA damage, lipid peroxidation, and free radical formation [171]. In addition, lithium induces BDNF in neuronal cultures [172].

### 8.3. ROS Scavengers

A mechanism that implicates the oxidative stress in the appearance of depressive symptoms is the metabolism of the nitric oxide (NO). NO is generated by nitric oxide synthase (NOS) that catalyzes the metabolism of L-arginine to L-citrulline and nitric oxide (∙NO) in the presence of molecular oxygen and NADPH. NO is a gas that acts as second messenger in a number of organs, including the brain, and is also a free radical involved in the etiology and progression of many diseases.

Neuronal NOS (nNOS) is a Ca2+-calmodulin-dependent isoform of NOS that is constitutively expressed in neuronal cells. A persistent expression of nNOS may result in an increased production of reactive nitrogen species (RNS), such as ∙NO and peroxynitrite (ONOO^−^) and thus may result in neuronal death due to increased nitrative/nitrosative stress. NOS can also generate superoxide when levels of the natural substrates L-arginine and tetrahydrobiopterin decrease [173].

The “NO hypothesis” was tested in a small clinical study done on 78 patients diagnosed with recurrent depressive disorder and healthy controls. High levels of plasma NO were tested along with the efficiency of visual-spatial and auditory-verbal working memory and short-term declarative memory. The concentration of plasma NO was found to be directly proportional with the severity of depressive symptoms [174].

In a recent study, Yoshitomi and colleagues [175] have reported the synthesis of pH-responsive nitroxide radical-containing nanoparticles which act as highly efficient scavengers of ROS, thus bringing new hopes for antioxidant therapies.

Recently it is has been suggested that not only antioxidants, but also the prooxidant system plays an important role in neuropsychiatric disorders. Xanthine oxidase (XO) is an enzyme of special interest in this context, since it acts as a prooxidant, but its main product uric acid is a powerful antioxidant. By examining the activity of XO in the occipital cortex and thalamus of patients with psychosis, the authors found a decreased activity of XO suggesting a downregulation of cellular defence mechanisms in schizophrenia [176].

The available drugs currently in use either for research or therapeutic purposes for several brain diseases are shown in Table 1.

## 9. Conclusions

Here we reviewed many aspects of therapeutic strategies aimed to improve the neuroprotection and the function of the brain. Many preclinical models showed an increased neuroprotection to stressors like hypoxia. Although many promising drugs, in particular antioxidants, have been developed and shown to be beneficial to experimental animal models, the results of recent clinical trials investigating these promising drugs have been largely negative. As alluded to previously, the oxidative stress is a key contributor to neurodegeneration. Therefore, the antioxidant therapy is a novel therapeutic strategy and neuroscientists are increasingly interested in the participation of ROS towards the pathology involved in neurodegenerative disorders. It is, however, difficult to determine targets for treatment and to distinguish between what may be harmful or beneficial for the brain, without precise knowledge of the pathways involved in the progression of neuronal diseases [178].

Modulation of prooxidant-antioxidant balance plays an important role in mitochondrial dysfunction and provides an additional therapeutic option which can be used to improve neuroprotection and cognitive functions in response to oxidative stress. Anti-ROS drugs could probe pathological pathways associated with neurodegeneration psychiatric disorders. However, there are only few well-established drugs of such a kind and the risks and benefits are still not fully clarified. Most likely, multiple distinct pathways should be targeted for an efficient therapeutic purpose. Therefore it is not surprisingly that many drugs exert their beneficial effects on neurotransmission indirectly by modulating inflammation, oxidative stress, or apoptosis [179]. Meta-analyses have suggested that antidepressants (fluvoxamine, reboxetine, or imipramine) and antipsychotics (clozapine and risperidone) reduce the levels of the proinflammatory cytokines IL-6 and NO and suppress the macrophage production [177] and upregulate signaling pathway associated with neurotrophic factors like BDNF [180, 181]. Likewise, fluoxetine may exert its neuroprotective effects via downregulating the expression of inhibiting key players involved in inflammation including NFkappaB [182], IL-1*β*, TNF-*α*, and COX-2 [183]. However, the available drugs have pleiotropic actions and are not fully characterized in the clinic.

## Figures and Tables

**Table 1:** Drugs with anti-inflammatory and antioxidant effects currently under investigation for the treatment of several neuropsychiatric diseases.

Drug	Brain disease	Mechanism	Reference
*ω*-3 PUFA	ADHD	Nonspecific antioxidant effect	[151, 152]
Vitamin C	Vascular dementia	Free radical scavenger	[153]
Hyperbaric O_2_	Autism	Anti-inflammatory and reduces oxidative stress	[157]
H_2_S	Focal cerebral ischemia/reperfusion	Inhibits oxidative phosphorylation, inflammation, and apoptosis	[159]
H_2_-rich saline	Focal or global cerebral ischemia/reperfusion	Reduces oxidative stress and inflammation	[160, 161]
Lithium	Healthy volunteers	Prevents/or reverses DNA damage, lipid peroxidation, and free radical formation	[164]
Pifithrin-*μ*	Perinatal hypoxic-ischemic brain damage	Inhibition of the apoptotic pathways and reduction of the oxidative stress	[166]
Telmisartan	Vascular dementia	Anti-inflammatory and antioxidant effects	[174–176]
Nanodrugs		ROS scavengers	[177]
Triterpenoids	Parkinson	Antioxidants via Nrf2/ARE pathway	[146]
PPARgamma agonists	Neurodegenerative diseases	Anti-inflammatory	[162–164]
